# SMAD4 Regulates Cell Motility through Transcription of N-Cadherin in Human Pancreatic Ductal Epithelium

**DOI:** 10.1371/journal.pone.0107948

**Published:** 2014-09-29

**Authors:** Ya'an Kang, Jianhua Ling, Rei Suzuki, David Roife, Xavier Chopin-Laly, Mark J. Truty, Deyali Chatterjee, Huamin Wang, Ryan M. Thomas, Matthew H. Katz, Paul J. Chiao, Jason B. Fleming

**Affiliations:** 1 Department of Surgical Oncology, The University of Texas M.D. Anderson Cancer Center, Houston, Texas, United States of America; 2 Department of Molecular and Cellular Biology, The University of Texas M.D. Anderson Cancer Center, Houston, Texas, United States of America; 3 Department of Gastroenterology and Rheumatology, the Fukushima Medical University School of Medicine, Fukushima, Japan; 4 Clinique Charcot, Sainte Foy lès Lyon, France; 5 Department of Surgery, Mayo Clinic, Rochester, Minnesota, United States of America; 6 Department of Pathology and Immunology, Baylor College of Medicine, Houston, Texas, United States of America; 7 Department of Pathology, the University of Texas MD Anderson Cancer Center, Houston, Texas, United States of America; 8 Department of General Surgery, the University of Florida College of Medicine, Gainesville, Florida, United States of America; University of Birmingham, United Kingdom

## Abstract

Expression of the cellular adhesion protein N-cadherin is a critical event during epithelial-mesenchymal transition (EMT). The SMAD4 protein has been identified as a mediator of transforming growth factor-β (TGF-β) superfamily signaling, which regulates EMT, but the mechanisms linking TGF-β signaling to N-cadherin expression remain unclear. When the TGF-β pathway is activated, SMAD proteins, including the common mediator SMAD4, are subsequently translocated into the nucleus, where they influence gene transcription via SMAD binding elements (SBEs). Here we describe a mechanism for control of *CDH2*, the gene encoding N-cadherin, through the canonical TGFβ–SMAD4 pathway. We first identified four previously undescribed SBEs within the *CDH2* promoter. Using telomerase immortalized human pancreatic ductal epithelium, we found that TGF-β stimulation prompted specific SMAD4 binding to all four SBEs. Luciferase reporter and SMAD4-knockdown experiments demonstrated that specific SMAD4 binding to the SBE located at −3790 bp to −3795 bp within the promoter region of *CDH2* was necessary for TGF-β-stimulated transcription. Expression of N-cadherin on the surface of epithelial cells facilitates motility and invasion, and we demonstrated that knockdown of SMAD4 causes decreased N-cadherin expression, which results in diminished migration and invasion of human pancreatic ductal epithelial cells. Similar reduction of cell motility was produced after *CDH2* knockdown. Together, these findings suggest that SMAD4 is critical for the TGF-β-driven upregulation of N-cadherin and the resultant invasive phenotype of human pancreatic ductal epithelial cells during EMT.

## Introduction

The transition of epithelial cells to a mesenchymal phenotype (EMT) is a fundamental characteristic of carcinoma cells [1]. A lineage tracing study using genetically engineered mouse models of pancreatic adenocarcinoma demonstrated that EMT of pancreatic epithelial cells leads to their migration into surrounding stroma and entry into the bloodstream. Importantly, these events were observed before the formation of a solid tumor in the animals [2]. These data suggest that seeding of distant organs occurs before pancreas tumor formation, an observation whose clinical relevance is supported by the high rate of metastasis experienced by patients with pancreatic cancer [3]. In humans, pancreatic inflammation is strongly associated with the subsequent development of pancreatic cancer. The animal lineage tracing study found that inflammation in the form of pancreatitis increased EMT and subsequent dissemination into the bloodstream [2]. Therefore, observations in both mouse models and patients identify inflammation-related EMT of pancreatic epithelial cells as an outcome-determining event in pancreatic cancer.

A major constituent of this process is the interaction between the pleiotropic cytokine transforming growth factor-β (TGF-β) and cadherins, which are transmembrane glycoproteins that mediate calcium-dependent cell–cell adhesion. TGF-β, an abundantly studied inducer of EMT, has been shown to regulate tissue homeostasis and prevent tumorigenesis. TGF-β dimers bind to TGF-β type II receptors, which phosphorylate TGF-β type I receptors via serine/threonine kinase activity, which in turn phosphorylate cytoplasmic SMAD2 and SMAD3. The phosphorylated SMAD protein then binds to SMAD4, which is subsequently translocated into the nucleus. The complex then binds gene promoter regions termed SMAD-binding elements (SBEs) in order to regulate transcription. Jonk et al reported the identification of SBEs composed of the sequence CAGACA in the promoter of the JunB gene, which is potently induced by TGF-β and the related cytokines activin and bone morphogenic protein (BMP) [4]. Others also identified the 8-bp palindromic sequence GTCTAGAC as a SBE [5]–[7]. TGF-β signaling can also be transduced through a non-canonical pathway, such as the ERK, JNK, and MAPK pathways, as well as some small GTPase pathways [8], [9].

SMAD4 is also considered a tumor suppressor gene that was originally recognized as “deleted in pancreatic carcinoma locus 4” (DPC4) on chromosome 18q21.1 [10], [11]. As a tumor suppressor, SMAD4 has been extensively analyzed, but reports of its function in EMT have been contradictory. SMAD4/DPC4 protein functions are required in the regulation of TGF-β–inducible EMT, which plays an important role in embryogenesis, cell adhesion, cellular motility, and cancer cell invasion and metastasis [12]–[15].

One characteristic phenotypic change of EMT is the upregulation of N-cadherin. The gene that encodes for N-cadherin, *CDH2*, is a classic gene of the cadherin superfamily. This gene is expressed mainly in mesenchymal cell types, including nerve tissues, myocytes, and fibroblasts [16], [17]. Expression of N-cadherin has been reported to enhance the invasive capacity, cell migration, metastasis, and angiogenesis of a variety of cancers, including those of the bladder, breast, esophagus, and thyroid [18]–[20]. Knockdown of N-cadherin in the BxPC-3 pancreatic cancer cell line was shown to lead to decreased tumor size and metastases in an orthotopic animal model [21]. These studies suggest that N-cadherin plays an important role in cancer metastasis and that understanding its regulation and function could help us to understand better of SMAD4/N-cadherin related cell motility, and may explain mechanism of pancreatic tumor metastasis.

As TGF-β induces EMT and the TGF-β signaling pathway is transduced by SMAD4, our study has focused on how the SMAD4 restrains N-cadherin expression in human pancreatic ductal epithelium, we hypothesize that SMAD4, through the binding of SBEs, regulates N-cadherin expression and cell invasion and migration.

## Materials and Methods

### Ethics statement

All patients in the study signed written informed consent prior to undergoing planned pancreatectomy. Each patient was given the opportunity to either consent or decline to acquisition and storage of excess tumor tissue to be used in the patient-direct xenograft program. The protocol was approved by The University of Texas MD Anderson Cancer Center Institutional Review Board under #LAB07-0854. Excess patient tumor was collected only after the planned surgical resection and pathologic examination were complete. Three patient specimens from pancreatectomies performed between 2009 and 2011 at The University of Texas MD Anderson Cancer Center were selected after reviewing medical records and tissue specimens.

### Cell lines and reagents

Human pancreatic nestin-expressing (HPNE) cells, an hTERT-immortalized normal human ductal progenitor cell line [22], and 293T cells were obtained from Dr. Paul J. Chiao at The University of Texas MD Anderson Cancer Center at Houston, Texas. 293T and PANC-1 cell lines were obtained from ATCC (Manassas, VA). The established HPNE, PANC-1, and 293T cells were verified by DNA fingerprinting at the Characterized Cell Line Core Facility of the MD Anderson Cancer Center.

HPNE, PANC-1, and 293T cells were maintained in Dulbecco's modified Eagle's medium (Invitrogen Life Technologies, Grand Island, NY) supplemented with 10% fetal bovine serum (Invitrogen Life Technologies) at 37°C in a 5% CO_2_ environment. Human recombinant TGF-β was purchased from R&D Systems (#240-B, Minneapolis, MN).

### Plasmid transfection SMAD4

Human retroviral short hairpin RNAi against human SMAD4 sequence 5′-GGTGTGCAGTTGGAATGTA-3′ (shSMAD4) was identified from the He et al. article [23], and the first 4 base pairs were replaced by AAAA and used as a scrambled control SMAD4 short hairpin RNA (shScr). Its sequence was 5′-AAAATGCAGTTGGAATGTA-3. The pRetrosuper-GFP shSMAD4 plasmid was purchased from Addgene (plasmid 15724; Cambridge, MA). shSMAD4 and shScr recombinant viruses were generated by transient transfection of the packaging plasmids pMLg/pRRE, pRSV.rev, and pHCMV-G into 293T cells. Virus-containing supernatant was collected after 72 hours to infect HPNE cells. The infected HPNE cells were purified by GFP fluorescence-activated cell sorting at the Flow Cytometry and Cellular Imaging Facility of MD Anderson. The effectiveness of SMAD4 knockdown was confirmed by western blotting and reverse-transcriptase (RT) polymerase chain reaction (PCR) analysis.

### Western blotting and immunofluorescence staining

HPNE cells were harvested and solubilized in radioimmunoprecipitation assay protein lysis buffer (50 mM Tris HCL at pH 7.4, 150 mM NaCl, 1% Nonidet P-40, 0.5% sodium deoxycholate, 0.1% SDS, 0.1 mM EDTA, 1 mM sodium orthovanadate, 1 mM NaF, and 1x protease inhibitor cocktail) (Roche, Indianapolis, IN). Cell lysates (20 µg) were separated by electrophoresis on 8–10% SDS polyacrylamide gels, transferred to PVDF membranes (Millipore, Billerica, MA), and probed with different dilutions of antibodies of interest. The antibodies used in this study were against phospho-SMAD2 (Ser465/467), phospho-SMAD3 (Ser423/425), phospho-Akt (Ser473), phospho-MEK1/2 (Ser217/221), SMAD4, Tak1, SMAD2/3, MEK1/2, p21 Waf1/Cip1 (all from Cell Signaling Technology, Danvers, MA), N-cadherin, vimentin, and cytokeratin 19 (CK19) (from Abcam, Cambridge, MA). Actin for protein loading control was purchased from Sigma-Aldrich (St. Louis, MO). Reactive bands were visualized with enhanced chemiluminscent reagents (GE Healthcare, Piscataway, NJ).

For TGF-β stimulation and phosphorylation studies, 90% confluent HPNE cells were washed twice with PBS and kept in serum-free medium overnight. Fresh 5% FBS medium was then added and the cells were incubated with recombinant TGF-β (5 ng/ml) for 2, 8, or 24 hours. Cell lysates were collected and immunoblotting with phospho-specific antibodies was used to analyze the various signaling pathways.

For immunofluorescence staining, HPNE cells were seeded into 8-well Lab-Tek II chamber slides (Thermo Fisher Scientific, Rochester, NY), fixed with 1% formalin the next day for 10 minutes at room temperature, and permeabilized with 1% Triton X-100 for 10 minutes. The chamber slides were then blocked with 1% BSA, and anti-N-cadherin and anti-CK19 antibodies (5 ng/µl) were added and incubated overnight at 4°C. Secondary antibodies conjugated with Alexa Fluor 488 or Rhodamine Red-X (2 ng/µl) (Invitrogen Life Technologies, Grand Island, NY) were incubated with the slides for 30 minutes at room temperature, and the slides were counterstained with 4,6-diamidino-2-phenylindole (Sigma-Aldrich, St. Louis, MO for 10 minutes. Fluorescent mounting medium (Dako, Carpinteria, CA) was then added onto the slides, which were sealed with a cover glass. Immunofluorescence images were captured with an Olympus U-RFL-T fluorescent microscope (Center Valley, PA) using the same exposure time for all samples. As a negative control, HPNE cells were incubated with diluted buffer instead of primary antibody.

### RT-PCR and real-time PCR

Total RNA was extracted using TRIzol reagent (Invitrogen Life Technologies) based on standard procedures. Complementary DNA was prepared with the iScript reverse transcription supermix kit (Bio-Rad Life Science, Hercules, CA) according to the manufacturer's instructions. The primers were used in this study are listed in Table 1. The expression level of human *CDH2* mRNA was quantified using iQ SYBR Green Supermix (Bio-Rad Life Science). Relative expression level was determined by normalizing the expression level of each target to glyceraldehyde 3-phosphate dehydrogenase (GAPDH), and relative mRNA fold change was determined using the 2^(−ΔΔCt)^ method. Amplification was conducted with a total volume of 10 µl for 40 cycles of 30 seconds at 94°C, 30 seconds at 55°C, and 30 seconds at 72°C. Samples were run in triplicate. Three independent experiments were performed. Statistical significance was tested by the two-tailed *t*-test at *P* values <0.05.

**Table 1:** Primers used in this study for RT-PCR or real time PCR.

Gene	Forward (5′-3′)	Reverse (5′-3′)	Amplicon (bp)
***CDH1***	tgcccagaaaatgaaaaagg	gtgtatgtggcaatgcgttc	200
***CDH2***	ggacagttcctgagggatca	ggattgccttccatgtctgt	253
***VIM***	ggctcagattcaggaacagc	gcttcaacggcaaagttctc	327
***BCAT***	atgggatcaaacctgacagc	cagatctcttggccctcaac	220
***FN1***	cgagcttccccaactggtaaccc	agcttcttgtcctacattcggcgg	277
***TWIST1***	acgagctggactccaagatg	cacgccctgtttctttgaat	291
***TWIST2***	agagcgacgagatggacaat	gcatctctgtcctgggttgt	327
***ZEB2***	gccttgagtgctcgataagg	ttcctgggctacgaccatac	392
***GAPDH***	acggatttggtcgtattggg	tgattttggagggatctcgc	200

### Modified Boyden chamber invasion and migration assays

A modified Boyden invasion chamber assay was used as previously described [24], [25]. HPNE cells transfected with shSMAD4 (HPNE/shSMAD4 cells) and HPNE cells transfected with shScr (HPNE/shScr cells) were seeded (5×10^4^ cells) onto serum-free medium in the top compartment of Matrigel-coated chambers (8.0-µm pores, BD Biosciences, Bedford, MA), where these cells were incubated with or without human TGF-β (10 ng/ml), and 20% FBS medium was added to the bottom compartment as a chemoattractant. Cells were allowed to invade across coated inserts for 20 hours. The cells on the apical surface of the insert were scraped off, and membranes with invaded cells were fixed in 100% methanol, stained with 1% crystal violet (Sigma-Aldrich), and mounted on slides. Invading cells were counted at 10x magnification in brightfield on an Olympus BX51 microscope in three different fields per membrane, and both the peripheral and the central areas were evaluated. Experiments were duplicated for each condition and repeated three times.

Migration was demonstrated using 100-mm non-pyrogenic dishes [26]. Briefly, when HPNE/shScr and HPNE/shSMAD4 cells approached 100% confluence, the cell monolayer was scratched using a P200 micropipette tip. Cells were washed in PBS twice and incubated in 5% FBS medium for 20 hours with or without TGF-β (10 ng/ml) to allow them to migrate and close the wound. Three random images were taken at the time of the scratch and at 20 hours (Olympus IX71 inverted microscope, 4× magnification). The migration rate was determined as the ratio of distance of the wound's gap at 20 hours versus at 0 hours using Adobe Photoshop software (San Jose, CA).

### Electrophoretic mobility shift assay

The *CDH2* promoter fragment was examined for recognition by nuclear proteins by electrophoretic mobility shift assay. Nuclear protein was extracted from cells according to a protocol previously described [27]. Four oligo probes containing an SBE consensus binding sites were used (Table 2). The competitive binding experiment was performed with a 25-fold amount of oligonucleotides with wild-type and mutant SBE consensus binding sites. OCT-1 probe (5′-TGTCGAATGCAAATCACTAGAA-3) was used as a loading control. The super-shift experiments were performed with anti-SMAD4 antibody (#SC-7966×, Santa Cruz Biotechnology, Santa Cruz, CA). The four potential SBE sequences of the promoter and mutant oligos were purchased from Sigma (Sigma-Aldrich, Woodlands, TX).

**Table 2:** Primers used for CDH2 ChiP assay.

Primers	Forwar (5′-3′)	Reverse (5′-3′)	Tm (°C)	Amplicon (bp)
A	aatcccctacatccctggag	ttgctgttggagactttgtgc	59.99/60.04	238
B	tgcactctcaaactcccaga	ctccttgggagcctaacgta	59.84/59.84	290
C	cacaggcagaaagcattcaa	accctttcacctctcccagt	59.99/59.97	371

### Chromatin immunoprecipitation (ChIP)

ChIP assay was performed with a kit (EMD Millipore, Billerica, MA) as previously described [28]. Briefly, cells were cross-linked with 1% PFA, lysed, and sonicated, and the cell lysates were incubated with Protein A/G beads (Santa Cruz Biotechnology) with an anti-SMAD4 antibody (Cat #sc-7966X, Santa Cruz Biotechnology) overnight. DNA was reverse cross-linked and purified. Three real-time PCR primers (for *CDH2* promoter sites A, B, and C, respectively) were used to amplify the corresponding region in the *CDH2* promoter containing the three SBE binding sites. Primers used for real-time PCR are listed in Table 2.

### *CDH2* promoter luciferase reporter assay

The *CDH2* promoter Firefly luciferase reporter gene was cotransfected into HPNE and 293T cells with an internal control, TK-Renilla luciferase, by using Lipofectamin2000 (#11668, Invitrogen). These cells were treated with TGF-β at different time points after 48 hours of transfection, and the activities of Firefly and Renilla luciferase were determined using a dual luciferase reporter assay system (#E4550, Promega, Madison, WI). Firefly luciferase activity was normalized to the Renilla luciferase activity of the internal control.

For site-directed mutagenesis assays, one base in the second SBE sequence in the *CDH2* promoter region from −3795 bp to −3790 bp was changed using a mutagenesis kit (# 200522, Agilent Technologies, Inc., Santa Clara, CA). This SBE sequence of CAGACA was mutated into CACACA. The experiments were performed in triplicate.

### Immunohistochemical staining

Tissues for this study were obtained from patients with primary pancreatic tumors and incorporated into our patient-direct xenograft program [29]. The tissues were embedded and cut into sections 4 µm thick for immunohistochemical study at the Clinical Core Laboratory of MD Anderson. Immunohistochemical staining for SMAD4 and N-cadherin was done using a Lab-Vision 480-2D immunostainer (ThermoFisher, Fremont, CA). All reactions were visualized with diaminobenzidine as a chromogen. Positive and negative controls were included in each run for each antibody used. Isotype controls for all antibodies were negative. Images were captured with an Olympus DP72 camera and CellSens software (Center Valley, PA) on an Olympus BX51 microscope at 10X magnification.

### Statistical analysis

All quantified data were plotted and analyzed using GraphPad Prism 5.0 software (La Jolla, CA) with Student's unpaired *t*-test or two-way ANOVA. Data are representative of at least 3 independent experiments and are reported as the mean ± SEM of replicates or triplicates unless otherwise indicated. A *P* value of <0.05 was considered statistically significant.

## Results

### Knocked down SMAD4 reduces N-cadherin protein level and inhibits invasion and migration in HPNE cells

**Figure 1:**
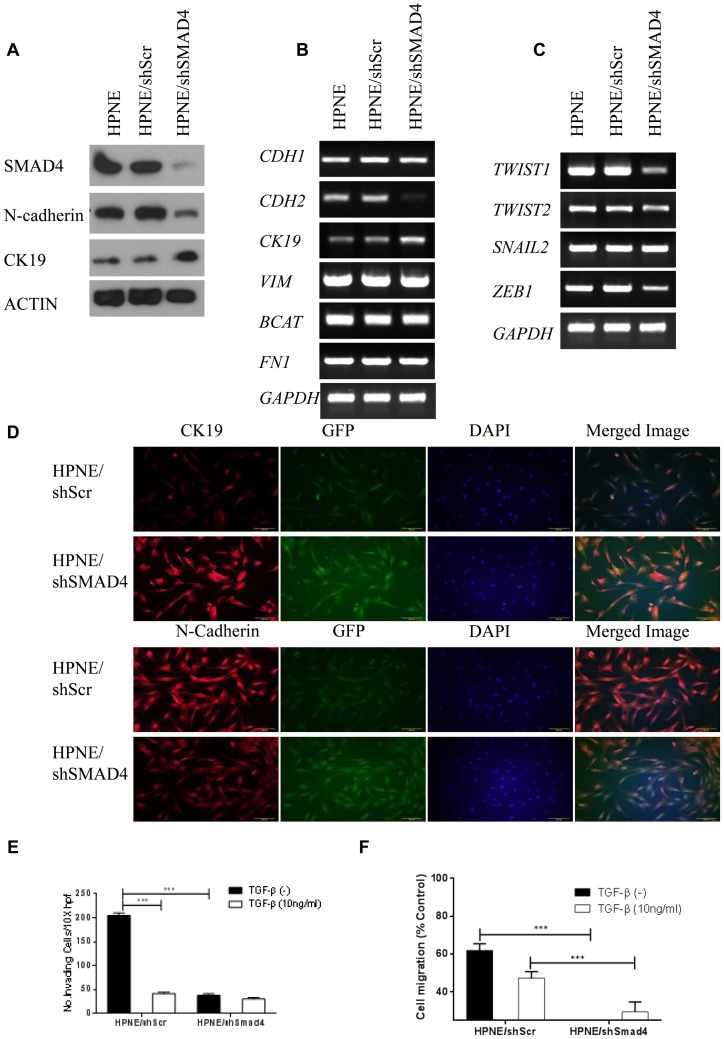
Knocked down SMAD4 reduces N-cadherin protein level and inhibits invasion and migration in HPNE cells. (a) Western blot analysis of cell expression of SMAD4 and N-cadherin and CK19. Actin was used as the loading control. (b) RT-PCR results showing cell mRNA levels of *CDH1, CDH2, CK19, VIM, BCAT*, and *FN1*, and (c) *TWIST1, TWIST2, SNAIL2*, and *ZEB1*. *GAPDH* was used as the housekeeping control. (d) Immunofluorescence staining of CK19 and N-cadherin in HPNE/shScr or HPNE/shSMAD4 cells. CK19 and N-cadherin were labeled with red fluorescent Alexa Fluor 594 goat anti-rabbit IgG (A11012, Invitrogen). GFP-positive cells represent cells transfected with shScr or shSMAD4. Nuclei were counterstained with blue fluorescent 4,6-diamidino-2-phenylindole. Images were merged using Olympus CellSens software. (e) Modified Boyden chamber assay. HPNE/shScr and HPNE/shSMAD4 Cells were added with or without 10 ng/ml TGF-β to serum-free media inserts in the top chamber, and 20% FBS was placed in the bottom chamber as a chemoattractant. Invasive cells were counted in 3 fields at 10× magnification in duplicated inserts. (f) Wound-healing assay. HPNE/shScr and HPNE/shSMAD4 cells were treated with or without10 ng/ml TGF-β. The y-axis represents cell migration distance at the time of the scratch and after 20 hours. Three random images (4×) were taken at these time points, and migration rate was determined as the ratio of distance at 20 hours versus 0 hours in the wound's gap using Adobe Photoshop software. Results are the mean ± s.d. of 3 independent experiments.

To assess the role of SMAD4 in TGF-β–mediated EMT in pancreatic ductal cells, we used shRNA to silence SMAD4 expression [30] in HPNE cells. RT-PCR, immunoblotting, and immunofluorescence analyses demonstrated a decrease in the level of the mesenchymal protein N-cadherin and an increase in the epithelial marker cytokeratin 19 (CK19) (Figure 1a, 1b and 1d). These results indicated that knock down of SMAD4 in HPNE cells not only down-regulated TGF-β-driven EMT, it also suggested a reversal of this process, known as mesenchymal-epithelial transition (MET) [14], [31]. The EMT-upregulating transcription factors *TWIST1* and *ZEB1* were also partially suppressed after SMAD4 knockdown (Figure 1c). In contrast, knock down of SMAD4 in HPNE cells had no notable effect on the protein level of the mesenchymal protein vimentin or the epithelial protein β-catenin (data not shown). It also had no effect on the mRNA levels of *CDH1, VIM, BCAT*, and *FN1*. Western blot results demonstrated that proteins from the canonical TGF-β pathway, phospho-SMAD2 and phospho-SMAD3, and non-canonical pathways, such as phospho-AKT, phospho-MEK 1/2, and TAK1 showed similar expression patterns after TGF-β treatment in HPNE, HPNE/Scr, and HPNE/shSMAD4 cell lines, suggesting they were unaffected (Figure S1a and 1b). p21, another downstream protein in the TGF-β pathway, showed the same pattern of expression in all three cell lines (Figure S1c). Cell proliferation assays indicated that these two cell lines have no significant cell growth alterations after exposure to TGF-β, as determined by MTT and colony formation assays (Figure S2a and S2b).

**Figure 2:**
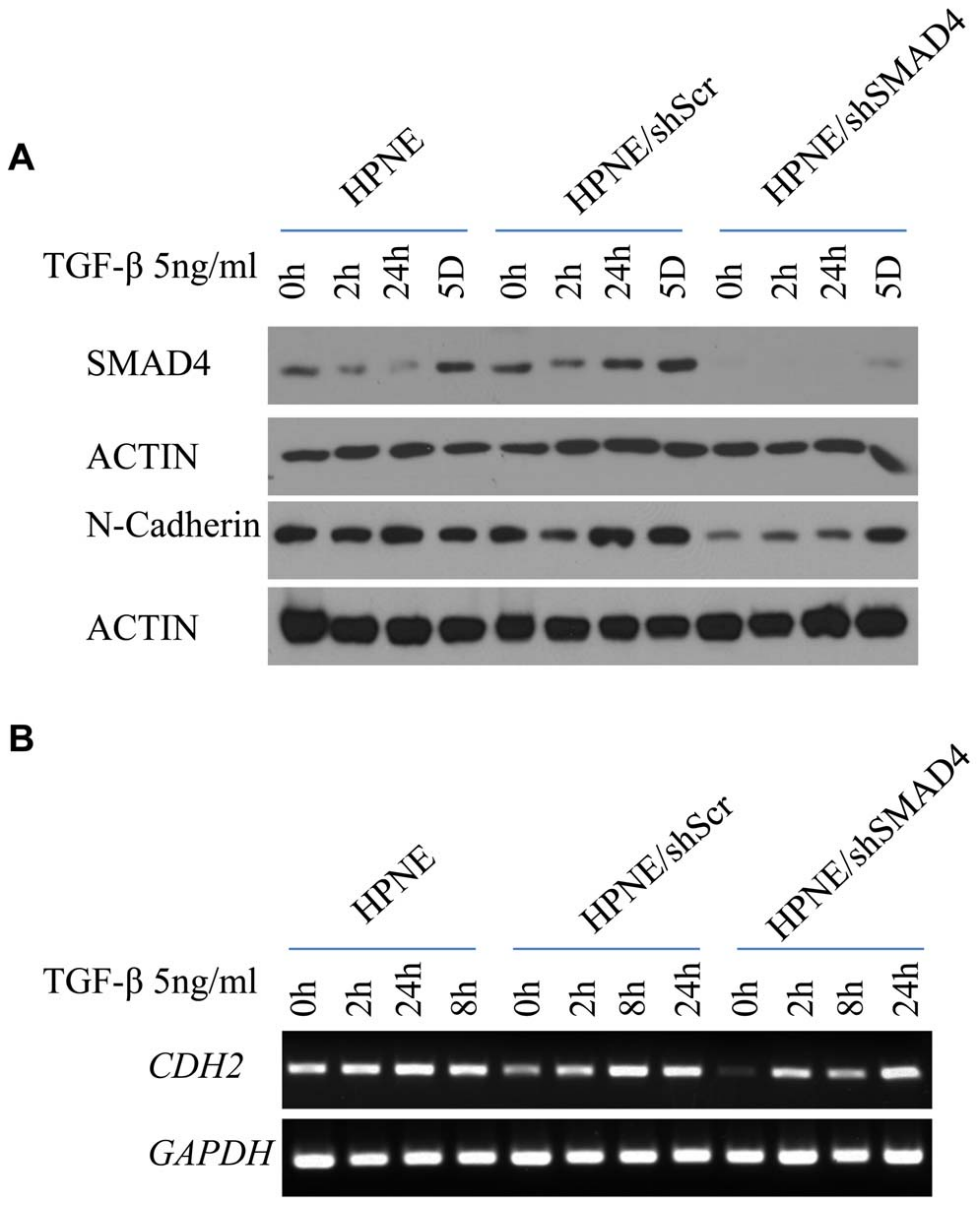
N-cadherin alteration after TGF-β treatment in HPNE, HPNE/shScr, and shSMAD4 cells. (a) Western blot analysis of cellular expression levels of N-cadherin and SMAD4 in cells treated with TGF-β (5 ng/ml) for 2 hours, 8 hours, 24 hours, or 5 days. Actin was used as the loading control. (b) *CDH2* mRNA level was measured by RT-PCR after TGF-β treatment (5 ng/ml) in 2 hours, 8 hours, and 24 hours' time points. *GAPDH* was used as the housekeeping gene control.

TGF-β–induced EMT is known to regulate the migration and invasion capacity of epithelial cells. Control HPNE/shScr cells were less invasive and less migratory after exposure to TGF-β (p<0.001). Both invasion and migration were greatly diminished after knockdown of SMAD4 in the HPNE/shSMAD4 cells (p<0.001) (Figure 1e and 1f). The effect of SMAD4 knockdown on TGF-β–induced N-cadherin expression was most evident after exposure of HPNE cells to TGF-β in culture. HPNE/shSMAD4 cells demonstrated lower protein levels of N-cadherin during short-term culture. However, SMAD4 and N-cadherin expression in HPNE/shSMAD4 cells was regained after continuous exposure to TGF-β over 5 days (Figure 2a). RT-PCR showed that *CDH2* in HPNE/shSMAD4 cells was regained quickly, in 2 hours after TGF-β treatment (Figure 2b). Taken together, these data suggested that SMAD4 is a necessary component of the TGF-β–driven expression of N-cadherin in HPNE cells and that N-cadherin expression contributes to the EMT phenotype.

### SMAD4 binds to SBEs located within the promoter region of *CDH2*

**Figure 3:**
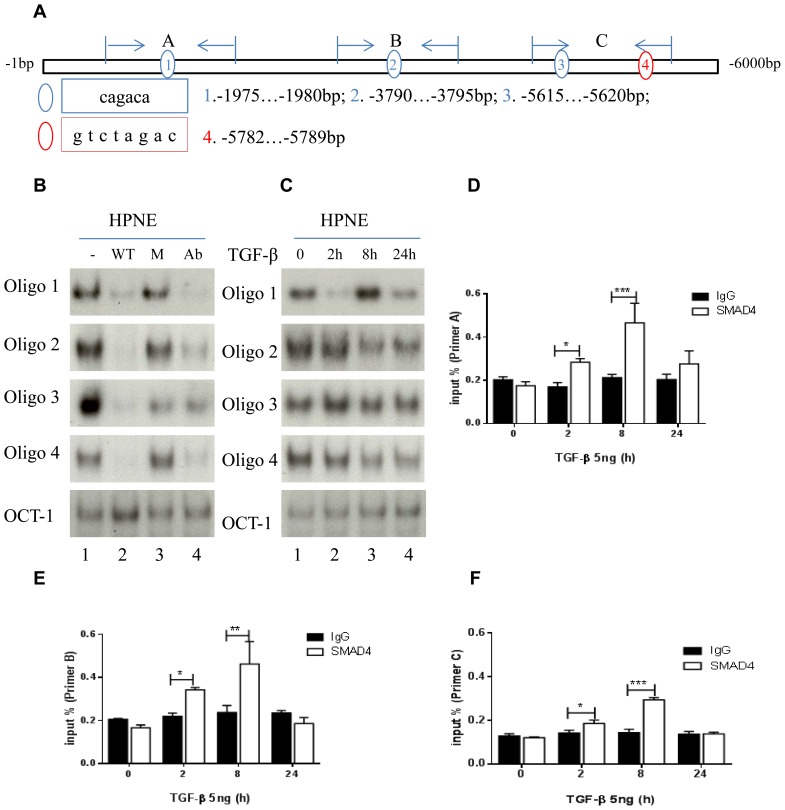
Map of multiple SBEs in *CDH2* promoter. (a) Three SBEs with a CAGACA sequence (blue circles 1, 2, and 3) and one SBE with a GTCTAGAC sequence (red circle 4). Sections A, B, and C represent 3 primers and an amplifying region for ChIP assay in the promoter. (b) Electrophoretic mobility shift assay results showed that 4 SBE oligos had strong DNA and nuclear protein interaction bands (lane 1), binding was quenched by wild-type (WT) oligos (lane 2) and not by mutant (M) oligos (lane 3), and anti-SMAD4 antibody (Ab) inhibited binding activity (lane 4). (c) SBE binding activity was regulated by TGF-β treatment at 0, 2, 8, and 24 hours. Oct-1 DNA binding activities were determined as loading controls in (b) and (c). (d-f) ChIP assays and real-time PCR of primers A, B, and C comparing the ratio of IgG to anti-SMAD4 antibody with or without 5 ng of TGF-β at 0 2, 8, and 24 hours. *P<0.05, **P<0.01, ***P<0.001.

On the basis of our preliminary observations (Figures 1 and 2), we therefore searched the region of the *CDH2* gene and identified four candidate SBE sequences located at three sites within the promoter (−1980, −3795, and −5620 bp). These sites may cooperate to regulate *CDH2* transcription (Figure 3a). Physical binding of SMAD4 to the sites was demonstrable by EMSA and ChIP assays. All four oligos have specific physical binding to HPNE nuclear protein. This binding was only blocked by wild type oligos but not by mutant oligos, and this binding was inhibited by a SMAD4 antibody *in vitro* (Figure 3a). The sequences of these wildtype and mutant oligos are listed in Table 3. When these cells were exposed to TGF-β, this binding was generally increased at 2 and 8 hours. These assays confirmed that exposure to TGF-β enhanced binding of the oligos to SBEs in the *CDH2* promoter (Figure 3c). ChIP assay results demonstrated that replicons from primers A, B, and C were increased at 2 hours (p<0.05) and 8 hours (p<0.01 and 0.001) after TGF-β treatment as determined by comparing signal from a SMAD4 antibody and an IgG control (Figure 3d, 3e, and 3f). Therefore, this ChIP assay corroborated the specific binding on the *CDH2* promoter as seen by EMSA. To check whether this binding can be generalized to pancreatic cancer cells, we used oligo 2 to perform EMSA in PANC-1 cells after TGB-β exposure. Results showed specific binding of oligo 2 to PANC-1 nuclear protein in a similar pattern as HPNE cells. This binding was decreased in 2 hours after TGF-β treatment. The binding was competed and inhibited by wildtype oligo 2 and SMAD4 antibody respectively, and not by mutant oligo 2 (Figure S3).

**Table 3:** Oligos from CDH2 promoter for EMSA.

Oligos	Wildtype sequence (5′-3′)	Mutant sequence (5′-3′)	bp
1	cgagcgcctcagacaacaatagctae	cgagcgcctaaaacaacaatagctae	26
2	cgagcgcctcagacaacaatagctag	tcttaaaacaagtaatgctttgagca	26
3	aaagcactttttttaattgcagacae	aaagcactttttttaattgcaaaaae	26
4	ttcctgtgaatactttgtctagactt	ttcctgtgaatactttaaaaagactt	26

### TGF-β stimulated CDH2 promoter dual-luciferase activity

**Figure 4:**
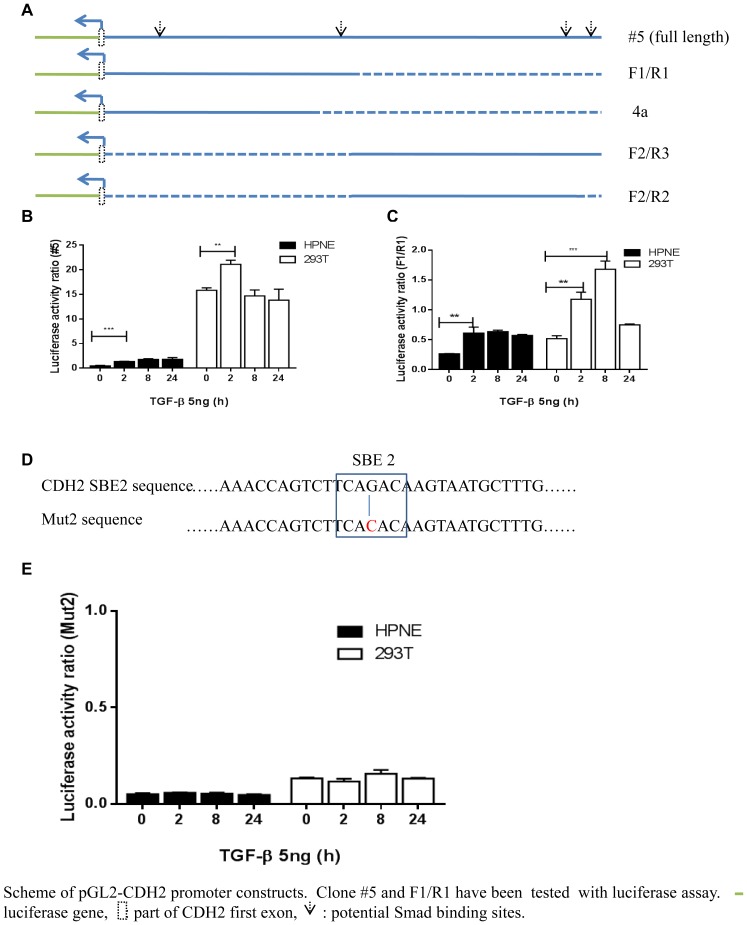
TGF-β Stimulated CDH2 promoter Dual-luciferase Activity. (a) Schematic of five pGL2-*CDH2* promoter constructs, including part of the *CDH* first exon (white rectangles), and potential SBE sites (downward arrows). (b,c) Luciferase activity ratios of *CDH2* clone #5 and F1/R1 construct treated with or without 5 ng of TGF-β at 0, 2, 8, and 24 hours in HPNE and 293T cells. **P<0.01, ***P<0.001. (d) The second SBE sequence in the *CDH2* promoter and the *Mut2* mutant sequence (G→C). (e) Luciferase activity ratio of *Mut2* with or without 5 ng TGF-β at 0, 2, 8, and 24 hours in HPNE and 293T cells.

Our results are the first to demonstrate that the presence of these SBEs is necessary for TGF-β–driven *CDH2* transcription. To validate that the SBEs within the *CDH2* promoter are necessary for TGF-β–induced transcription, we were utilized luciferase reporter constructs within HPNE and 293T cells. Five different constructs were cloned into pGL2-Basic vectors (Promega) (Figure 4a). Within HPNE cells, the highest levels of luciferase expression occurred when all four SBEs were intact (construct #5) suggesting that they all cooperate to enhance transcription of *CDH2*. Two SBEs (CAGACA) nearest to the *CDH2* coding region (construct F1/R1) were necessary for any expression to occur at baseline or after TGF-β stimulation in both HPNE and 293T cells. Luciferase activity was increased significantly at 2 hours with and without TGF-β treatment (construct #5, p<0.05) (Figure 4b), and again on construct F1/R1 at 2 hours and 8 hours (p<0.01 and 0.001; Figure 4c). The results suggested that the second SBE on construct F1/R1 was the most important for TGF-β stimulation of luciferase activity. The other three constructs (4a, F2/R3, F2/R2) produced no luciferase activity. To verify the importance of the second SBE on construct F1/R2, we specifically mutated the construct at −3790 from CAGACA to CACACA This caused luciferase activity to be significantly reduced with and without TGF-β treatment, thereby demonstrating that binding at this site is a vital step for *CDH2* transcription (Figure 4d and 4e).

### Loss of *CDH2* transcription and N-cadherin expression recapitulates the HPNE/shSMAD4 cell phenotype

**Figure 5:**
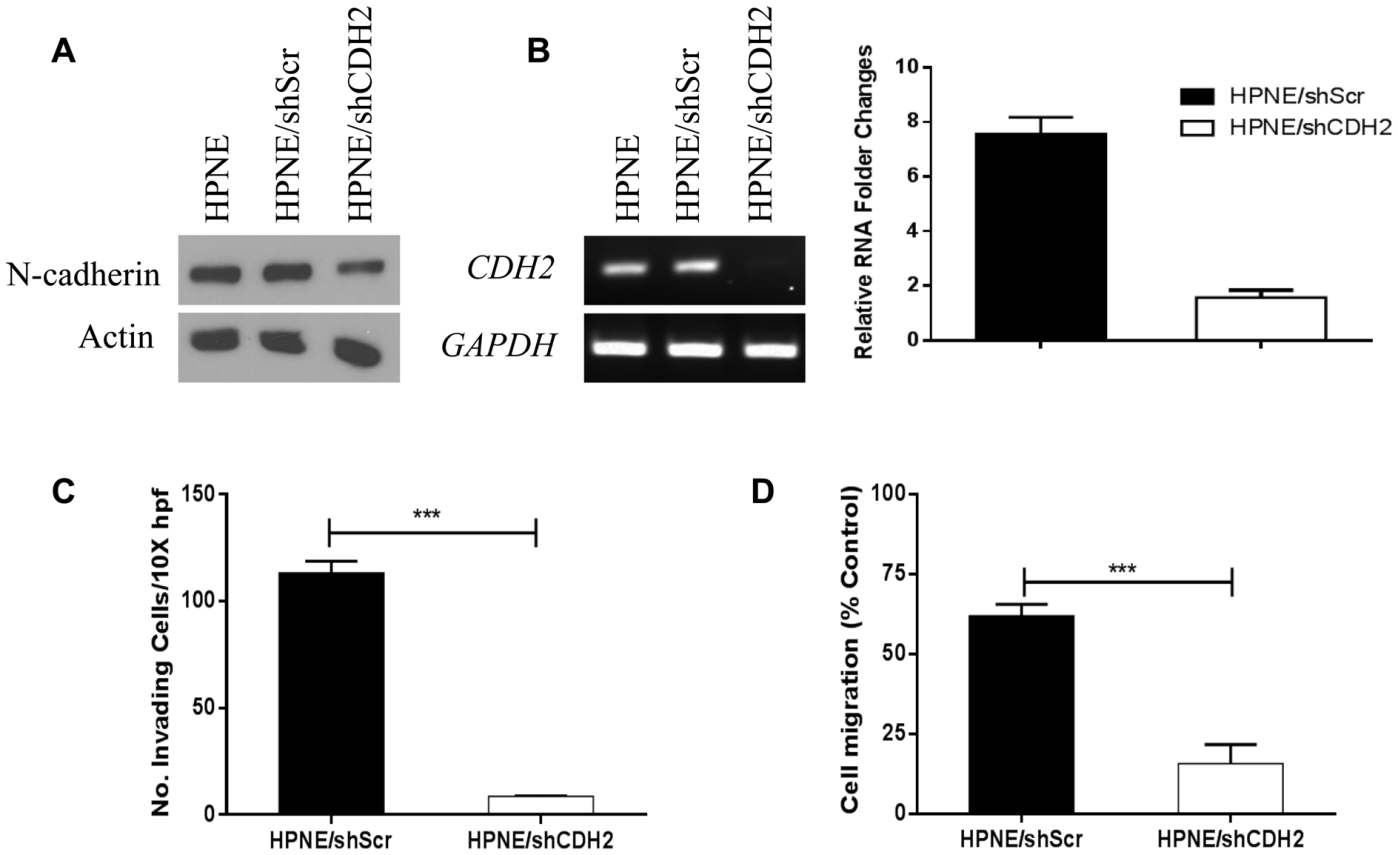
Invasion and migration assay after *CDH2* knockdown in HPNE, HPNE/shScr, and HPNE/shCDH2 cells. (a) N-cadherin was suppressed though shCDH2. Actin was used as the loading control. (b) RT-PCR (left) and real-time PCR (right) confirmed that *CDH2* mRNA was significantly decreased in HPNE shCDH2 cells. (c) Modified Boyden chamber assay was performed with transfected cells. Cells were treated with or without 10 ng/ml TGF-β in serum-free medium in the top inserts, and 20% FBS medium was used in the bottom chamber as a chemoattractant. Invasive cells were counted in 3 fields at 10× magnification in duplicated inserts. (d) Wound-healing assay was performed with transfected cells treated with or without 10 ng/ml TGF-β. Three random images (4× magnification) were taken at the time of the scratch (0 hours) and at 20 hours. Migration rate was determined as the ratio of the distance traveled at 20 hours versus 0 hours in the wound's gap using Adobe Photoshop software. **P<0.01, ***P<0.001.

We next sought to confirm whether loss of expression of the SMAD4-controlled target gene, *CDH2*, could produce the same cellular phenotype observed in SMAD4-knockdown HPNE cells. When sh*CDH2* was introduced to cells, production of N-cadherin and transcription of *CDH2* were inhibited as demonstrated by western blot (Figure 5a), and both RT-PCR (left) and real time PCR (right) (Figure 5b) in a pattern very similar to that observed in our preceding experiment. HPNE/shCDH2 cells demonstrated decreased invasive capacity and migration when compared to HPNE/shScr control cells (p<0.001) (Figure 5c and 5d).

### SMAD4 and N-cadherin immunohistochemistry staining on patient specimens

**Figure 6:**
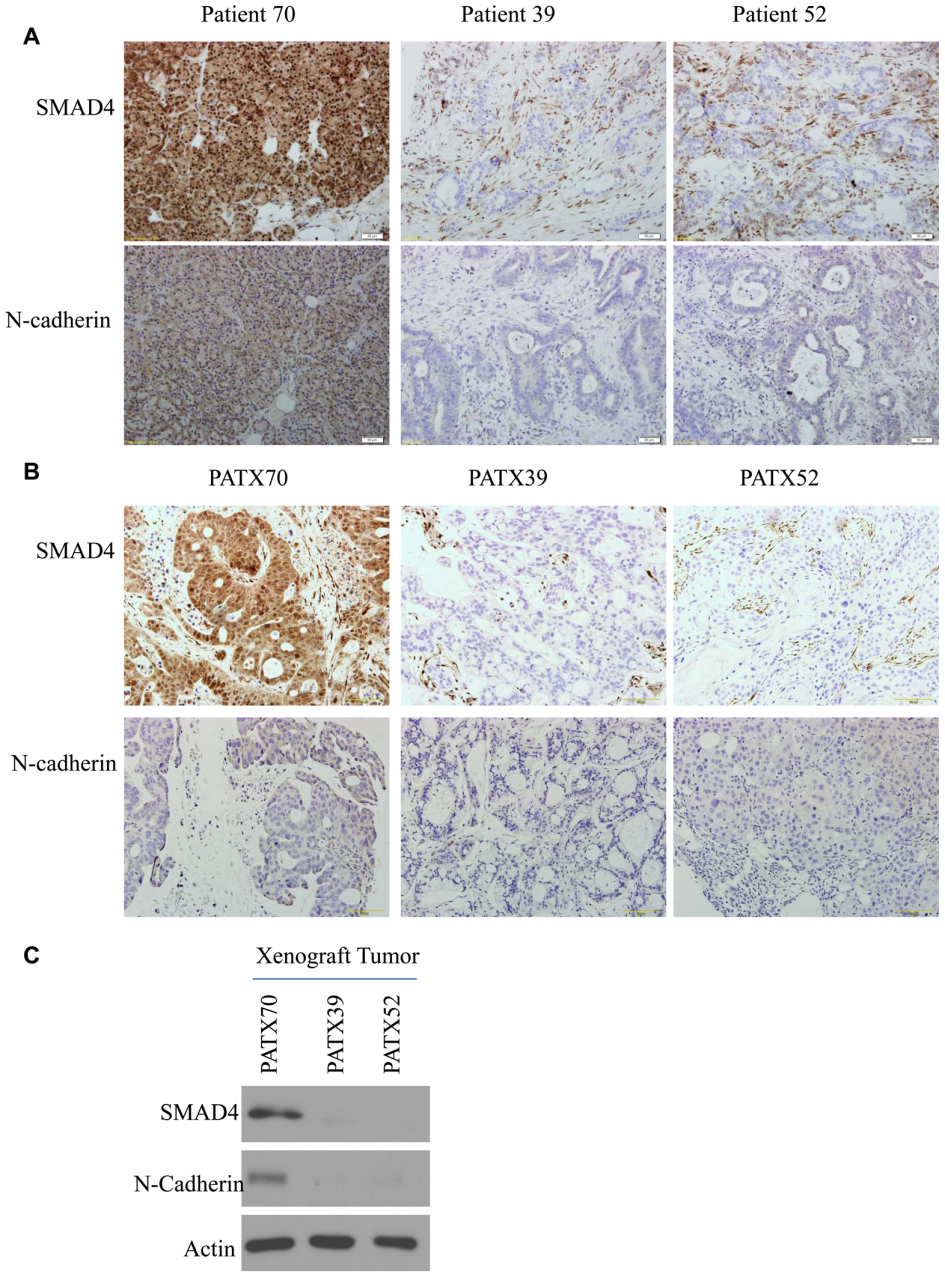
SMAD4 and N-cadherin expression levels in patient tumors and matched xenograft samples. (a) Representative immunostained images of patients' primary tumors and (b) and matched patient-direct xenograft tumors (PATX). Images were captured with an Olympus DP72 camera (10× magnification). (c) Western blotting results for corresponding patient-direct xenograft tumor lysates. Actin was used as the loading control.

We immunohistochemically stained specimens from 3 patients and matched xenograft samples and observed the correlation of SMAD4 and N-cadherin staining. The high expression of SMAD4 in tissue from patient 70 and in its derived xenograft PATX70 correlated with high N-cadherin expression, whereas the low expression of SMAD4 in tissues from patients 39 and 52 and in matched xenografts, PATX39 and PATX52, correlated with low expression of N-cadherin (Figure 6a and 6b). Western blotting was performed with lysates of these tumor xenografts, and similar correlations were observed (Figure 6c). The immunohistochemical results support our *in vitro* data that SMAD4 is important in maintaining N-cadherin expression, and that loss or mutation of SMAD4 can change a patient's local tumor environment through altering the N-cadherin level.

## Discussion

It is well known that EMT has a tremendous role in tumor invasion, metastasis, generating cancer stem cells, and drug resistance from *in vitro* and *in vivo* pancreatic cancer studies [32]. This phenotype is characterized by increased N-cadherin expression, whose functions have been extensively studied in EMT, tissue integrity, and cell proliferation [33], [34]. In this study, we have demonstrated previously unidentified SMAD4 SBEs located in the region of *CDH2* promoter, through which SMAD4 exerts transcriptional control over *CDH2*. These four candidate SBE sequences are located at three sites within the *CDH2* promoter (−1980, −3795, and −5620 bp). It is a novel finding that these sites may cooperate to regulate *CDH2* transcription. The *CDH2* promoter reporter gene assays revealed that SMAD4-dependent transcriptional activation is mediated by the SBEs, which indicates that *CDH2* is one of the downstream target genes regulated by SMAD4/DPC4.

The expression of N-cadherin is necessary for EMT and for the migration of epithelial cells [35]. We demonstrate that SMAD4 binding to these previously undescribed SBEs within the promoter region of *CDH2* is necessary for expression of N-cadherin on the surface of human pancreatic ductal epithelial cells. We have found that this mechanism of N-cadherin upregulation is necessary for human pancreatic ductal cells to migrate and invade through extracellular matrices, which would be features of metastatic cancer cells. These data contribute to our understanding of the complex genetic controls over EMT and metastasis. A limitation to this study is that the findings were demonstrated in only one non-cancerous pancreatic ductal cell line as well as one cancerous pancreatic ductal cell line. The fact that similar binding occurs in both cell lines suggests that this data can be generalized. However, further study is needed on how SMAD4 driven N-cadherin expression influences the clinicopathological features of human cancers [18]–[21].

In conclusion, these findings describe a TGF-β–SMAD4–N-cadherin transcription pathway that is necessary for the functional characteristics accompanying EMT in human pancreatic ductal epithelial cells.

## Supporting Information

Figure S1 **Changes in TGF-β canonical and non-canonical pathways in HPNE, HPNE/shScr, and HPNE/shSMAD4 cells.** Western blots of cells 2, 4, 8, and 24 hours after treatment with 5 ng/ml TGF-β. (a) Phospho-SMAD2 and phospho-SMAD3 protein expression levels. Total SMAD2/3 was used as the loading control. (b) Phospho-Akt, phospho-1/2Mek, and TAK1 protein expression levels. Respective loading controls were total Akt, 1/2 Mek, and actin. (c) p21 Protein expression levels. Actin was used as the loading control.
(TIF)

Figure S2 **Cell proliferation and colony formation assays of HPNE, HPNE/shScr, and HPNE/shSMAD4 cells.** (a) Cells were seeded at 1000 cells per well in 96 wells in triplicate and treated with fresh TGF-β (5 ng/ml) every other day. MTT assay was performed according to the manufacturer's recommendation. Absorbance was determined at 570 nm at days 1, 3, 5, and 7 using a microplate reader (FLUOstar Omega, BMG Labtech, Chicago, IL). (b) Cells were plated at 500 cells per 60-mm dish in triplicate and treated with fresh TGF-β (5 ng/ml) every other day and incubated for 14 days. The cells were then fixed and stained with 0.5% crystal violet in methanol. The number of colonies was counted manually.
(TIF)

Figure S3 **Electrophoretic mobility shift assay in PANC-1 cells.** (a) SBE oligo 2 had strong DNA and nuclear protein interaction bands (lane 1). Binding was quenched by wild-type (WT) oligo 2 (lane 2) and not by mutant (M) oligo 2 (lane 3). Anti-SMAD4 antibody (Ab) inhibited binding activity (lane 4). (b) SBE binding activity was regulated by TGF-β treatment at 0, 2, 8, and 24 hours. Free probe activities were determined as loading controls in (a) and (b).
(TIF)
