# Supplementary figures and images for: SMAD4 Regulates Cell Motility through Transcription of N-Cadherin in Human Pancreatic Ductal Epithelium
Source: PLoS One. 2014 Sep 29;9(9):e107948. doi: 10.1371/journal.pone.0107948 (PMC4180072; doi:10.1371/journal.pone.0107948)

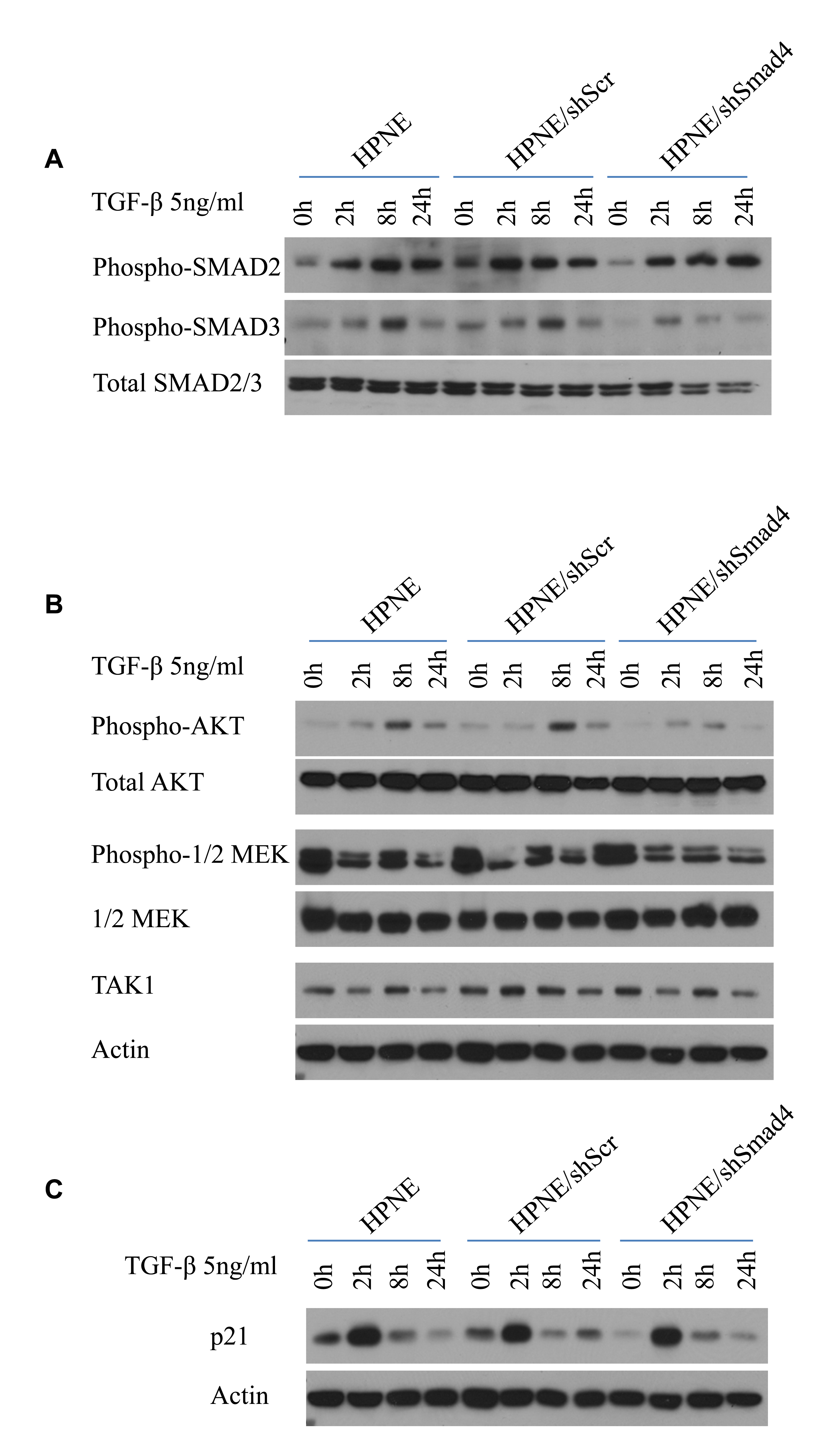

Supplement: Figure S1 — Changes in TGF-β canonical and non-canonical pathways in HPNE, HPNE/shScr, and HPNE/shSMAD4 cells. Western blots of cells 2, 4, 8, and 24 hours after treatment with 5 ng/ml TGF-β. (a) Phospho-SMAD2 and phospho-SMAD3 protein expression levels. Total SMAD2/3 was used as the loading control. (b) Phospho-Akt, phospho-1/2Mek, and TAK1 protein expression levels. Respective loading controls were total Akt, 1/2 Mek, and actin. (c) p21 Protein expression levels. Actin was used as the loading control. (TIF) [file pone.0107948.s001.tif]

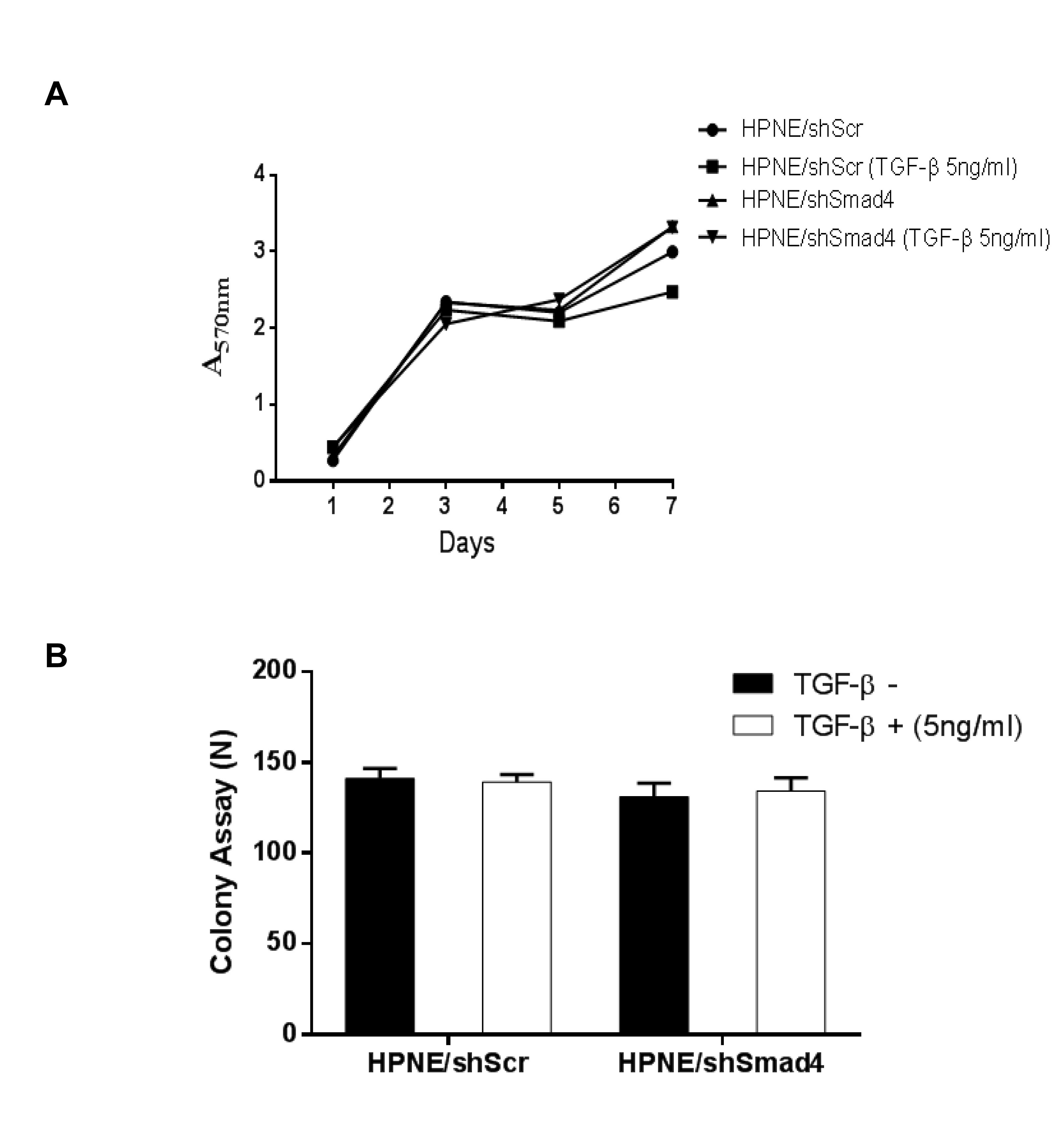

Supplement: Figure S2 — Cell proliferation and colony formation assays of HPNE, HPNE/shScr, and HPNE/shSMAD4 cells. (a) Cells were seeded at 1000 cells per well in 96 wells in triplicate and treated with fresh TGF-β (5 ng/ml) every other day. MTT assay was performed according to the manufacturer's recommendation. Absorbance was determined at 570 nm at days 1, 3, 5, and 7 using a microplate reader (FLUOstar Omega, BMG Labtech, Chicago, IL). (b) Cells were plated at 500 cells per 60-mm dish in triplicate and treated with fresh TGF-β (5 ng/ml) every other day and incubated for 14 days. The cells were then fixed and stained with 0.5% crystal violet in methanol. The number of colonies was counted manually. (TIF) [file pone.0107948.s002.tif]

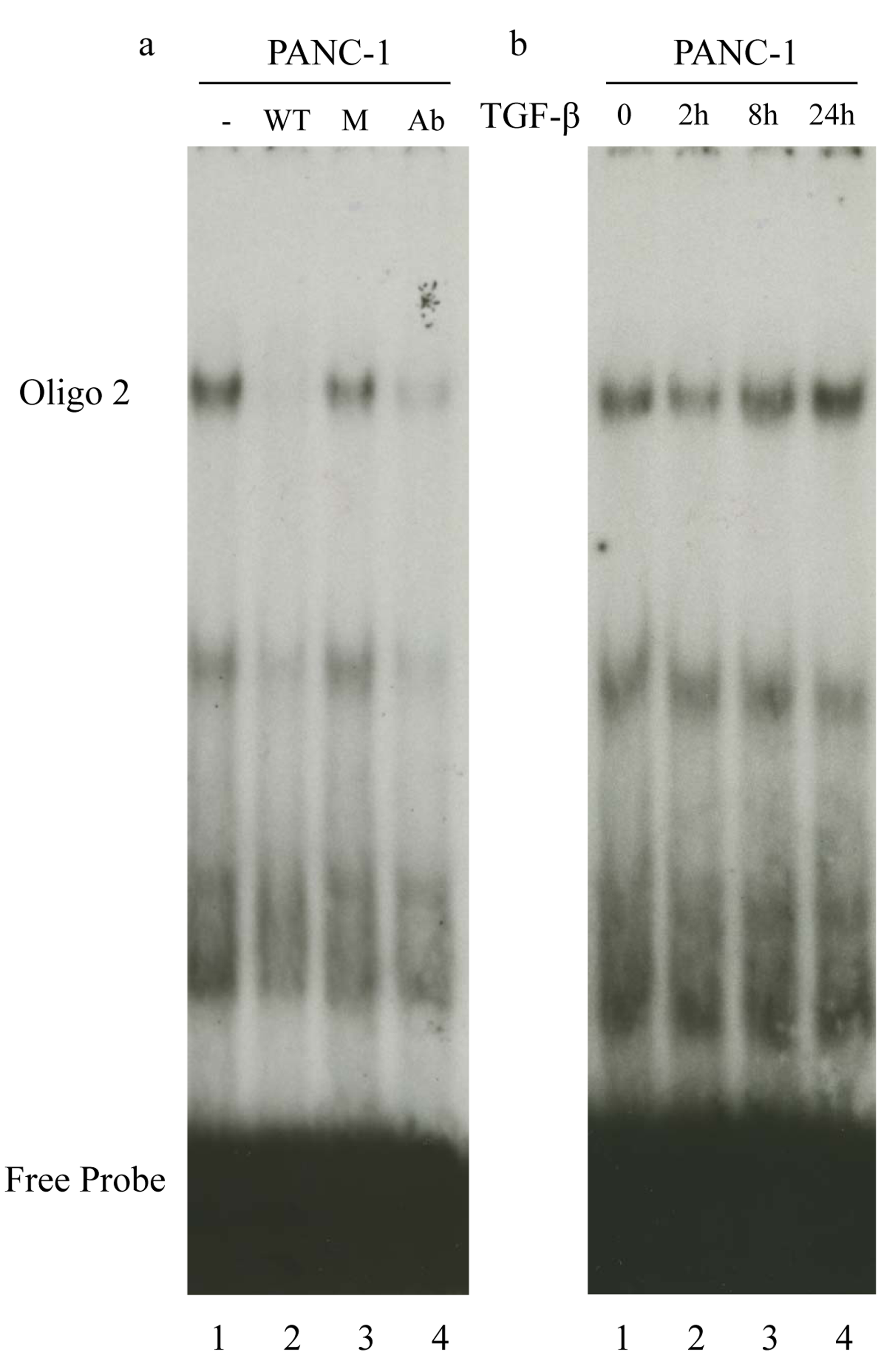

Supplement: Figure S3 — Electrophoretic mobility shift assay in PANC-1 cells. (a) SBE oligo 2 had strong DNA and nuclear protein interaction bands (lane 1). Binding was quenched by wild-type (WT) oligo 2 (lane 2) and not by mutant (M) oligo 2 (lane 3). Anti-SMAD4 antibody (Ab) inhibited binding activity (lane 4). (b) SBE binding activity was regulated by TGF-β treatment at 0, 2, 8, and 24 hours. Free probe activities were determined as loading controls in (a) and (b). (TIF) [file pone.0107948.s003.tif]
